# Productivity of sodic soils can be enhanced through the use of salt tolerant rice varieties and proper agronomic practices

**DOI:** 10.1016/j.fcr.2016.02.007

**Published:** 2016-04

**Authors:** Y.P. Singh, V.K. Mishra, Sudhanshu Singh, D.K. Sharma, D. Singh, U.S. Singh, R.K. Singh, S.M. Haefele, A.M. Ismail

**Affiliations:** a Indian Council of Agricultural Research-Central Soil Salinity Research Institute, Regional Research Station, Lucknow, India; b International Rice Research Institute, Delhi Office, India; c Indian Council of Agricultural Research-Central Soil Salinity Research Institute, Karnal, India; d International Rice Research Institute, Los Baños, Philippines; e Australian Centre for Plant Functional Genomics, University of Adelaide, Australia

**Keywords:** Crop management, Cost effective options, Nutrient management, Salt affected soils, Salt tolerant rice

## Abstract

Regaining the agricultural potential of sodic soils in the Indo-Gangetic plains necessitates the development of suitable salt tolerant rice varieties to provide an entry for other affordable agronomic and soil manipulation measures. Thus selection of high yielding rice varieties across a range of sodic soils is central. Evaluation of breeding lines through on-station and on-farm farmers’ participatory varietal selection (FPVS) resulted in the identification of a short duration (110–115 days), high yielding and disease resistant salt-tolerant rice genotype ‘CSR-89IR-8’, which was later released as ‘CSR43’ in 2011. Several agronomic traits coupled with good grain quality and market value contributed to commercialization and quick adoption of this variety in the sodic areas of the Indo-Gangetic plains of eastern India. Management practices required for rice production in salt affected soils are evidently different from those in normal soils and practices for a short duration salt tolerant variety differ from those for medium to long duration varieties. Experiments were conducted at the Indian Council of Agricultural Research-Central Soil Salinity Research Institute (ICAR-CSSRI), Regional Research Station, Lucknow, Uttar Pradesh, India during 2011 and 2013 wet seasons, to test the hypothesis that combining matching management practices (Mmp) with an improved genotype would enhance productivity and profitability of rice in sodic soils. Mmp were developed on-station by optimizing existing best management practices (Bmp) recommended for the region to match the requirements of CSR43. The results revealed that transplanting 4 seedlings hill^−1^ at a spacing of 15 × 20 cm produced significantly higher yield over other treatments. The highest additional net gain was US$ 3.3 at 90 kg ha^−1^ N, and the lowest was US$ 0.4 at 150 kg ha^−1^ N. Above 150 kg ha^−1^, the additional net gain became negative, indicating decreasing returns from additional N. Hence, 150 kg N ha^−1^ was considered the economic optimum N application rate for CSR43 in these sodic soils. Using 150–60–40–25 kg N–P_2_O_5_–K_2_O–ZnSO_4_·7H_2_O ha^−1^ in farmers’ fields grown to CSR43 produced an average of 5.5 t ha^−1^ grain. The results of on-farm evaluation trials of CSR43 showed that matching management practices (Mmp) increased yield by 8% over existing best management practices (Bmp) recommended by ICAR-CSSRI for sodic soils and by 16% over framers’ management practices; however, combining Mmp with CSR43 resulted in 35% higher yields over farmers’ current varieties and management. This approach of combining cost effective crop and nutrient management options and a salt-tolerant variety can maximize the productivity and profitability of sodic soils in the alluvial Indo-Gangetic plains and in neighboring salt-affected areas of the Ganges mega delta in South Asia.

## 1 Introduction

Salt affected lands are estimated at about 955 million ha worldwide (Szabolcs, 1994), afflicting 7% of the world’s total arable land (Flowers et al., 1997). The Indo-Gangetic region in India (21° 55′–32° 39′N and 73° 45′–80° 25′E; Singh et al., 2010) has about 2.7 million ha of salt affected soils, consisting mostly of centuries-old barren sodic soils with no land use opportunities (NRSA and Associates, 1996). These soils have been regarded as unfit for agriculture due to high pH (>8.5) and concentrations of soluble salts that produce alkaline hydrolysis products such as Na_2_CO_3_ and NaHCO_3_, together with sufficient exchangeable sodium to cause poor physical soil characteristics.

Grain yield of rice in salt affected soils is much lower because of its high sensitivity to salt stress (Flowers and Yeo, 1989, Gao et al., 2007, Ismail et al., 2007). Rice is exceptionally sensitive to salinity and sodicity at early seedling stage (Aslam et al., 1988, Aslam et al., 1993) and high yield losses have been observed because of high mortality and poor crop establishment. Modern high yielding varieties require considerable investment to ameliorate these soils to ensure reasonable yields, but this investment is beyond the capabilities of the resource-limited small holder farmers living off these salt affected areas. Increasing and sustaining yields in these areas will require a system that integrates salt tolerant varieties with effective and affordable crop and nutrient management practices. Chowdhury et al. (1993) observed that the number of seedlings hill^−1^ and plant spacing were important factors determining plant population per unit area for optimum nutrient uptake and for accessing sufficient light for photosynthesis, which ultimately determine grain yield. Plant mortality in sodic soils is high when young seedlings are transplanted, adversely affecting plant establishment and growth (Dargan and Gaul, 1974). Older seedlings survive better and establish earlier, but they produce less tillers and exhibit poor growth (Mandal et al., 1994). The effects of seedling age become more noticeable in short duration varieties (Chandrakar and Chandravanshi, 1988).

Sodic soils are inherently low in organic matter (<0.1%), and available N, and are more responsive to N application. These soils are more prone to N losses due to higher N volatilization caused by high pH, further aggravating N deficiency. Microbial activity, which influences N mineralization, is restricted by salt stress (Abrol et al., 1988, Liu and Kang, 2014, Wong et al., 2010). Therefore, the requirement of added N in sodic soils is higher than in normal soils and salt tolerant varieties appear to respond better to higher N application than sensitive varieties (Dixit and Patro, 1994, Meena et al., 2003).

Apparently, the lack of high yielding salt tolerant rice varieties, together with good management strategies specific for sodic soils are the main reasons for low and unstable productivity. High yielding salt tolerant varieties, like CSR10, CSR13, CSR23, CSR27, CSR36, Narendra Usar dhan3, which can tolerate sodicity of up to pH 9.8, were developed through conventional plant breeding and have made good impacts in salt-affected areas of India (Mishra et al., 1992). However, these varieties are of medium to long duration (130–140 days) and often do not fit well in the rice–wheat system predominating the Indo-Gangetic plains. Integrating farmers’ participatory varietal selection approach (FPVS) within the evaluation process during breeding strengthens and accelerates the selection of varieties with characteristics desired by farmers and expedites their adoption when released. Using this approach, field evaluation of salt-tolerant breeding lines was conducted through the network of the “Stress Tolerant Rice for Africa and South Asia (STRASA)” project for five years under varying sodic soil conditions in Uttar Pradesh, India, culminating in the release of the salt tolerant variety “CSR43” in 2011 (Singh et al., 2013, Singh et al., 2014).

Suitable management practices for salt affected soils are obviously different than in normal soils. Moreover, any management option for sodic soils developed using medium to long duration salt-tolerant varieties might not be suitable for a short duration variety like CSR43. This study reports on the evaluation and release of CSR43 and on developing suitable crop and nutrient management practices to ensure better establishment and higher productivity of this variety, with the hypothesis that this combination will help to improve productivity and profitability of rice in the sodic soils of the eastern Indo-Gangetic plains.

## 2 Materials and methods

### 2.1 Germplasm evaluation through participatory varietal selection

A set of 126 geographically and genetically diverse rice genotypes was screened through researchers’ managed on-station and on-farm trials during 2001. This set includes local genotypes, advanced salt tolerant breeding lines and salt tolerant high yielding varieties. From 2002 to 2005, FPVS (Paris et al., 2011) was employed during evaluation, and a set of 6 genotypes was selected from on-farm trials (Table 1). These genotypes were further evaluated, taking into account traits desired by men and women farmers, including grain yield and quality. A scale of 1–10 (1 being least preferred, 10 most preferred) was used to score and rank important traits. Preference scores for each genotype were calculated by subtracting the total negative votes from total positive votes, then dividing by the total votes. Through this process, the genotype CSR-89IR-8 consistently ranked first based on both farmers’ preference ranking and grain yield. From 2006 to 2008, the performance of this genotype was further evaluated in farmers’ managed trials and compared with popular high yielding varieties, then released as CSR43 (Singh et al., 2013, Singh et al., 2014) (Table 1).

### 2.2 Development of management practices for CSR43 through on-station trials

#### 2.2.1 Site characterization

Field experiments were conducted from 2011 to 2013 at the experimental farm of ICAR-CSSRI, Regional Research Station, Uttar Pradesh, India (26° 47′ 58″N, 80° 46′ 24″E, 120 m above MSL). The study site is representative of large areas of abandoned alkali soils in the Indo-Gangetic plains. The soil is Typic Natrustalfs, with surface soil (0–15 cm) pH (1:2 soil:water) of 8.9 and sub-surface (>15 cm) pH of >9.4. The soil presents physical and nutritional constraints to plant growth due to poor soil water and soil air characteristics caused by high bulk density (>1.5 g cm^−3^) and low infiltration rate (<2 mm day^−1^) (Sharma et al., 2006). Poor soil aeration in sodic soils restricts root development, respiration and soil microbial activities resulting in poor crop growth and productivity. Before the experiment, soil samples were collected from the 0–120 cm soil profile of the experimental field. The samples were air dried, ground in a Wiley mill and passed through a 2.0 mm sieve. The pH and EC of the soil were determined using digital pH and conductivity meters (Jackson, 1967). The pH and EC of surface soil (0–15 cm) were 9.2 and 0.61 dS m^−1^, respectively. Organic carbon was determined using the rapid titration method of Walkley and Black, modified by Walkley (1947) and was 0.29%. The concentration of Ca^2+^ + Mg^2+^, measured with an Inductivity Coupled Plasma (ICP) Analyzer (PerkinElmer), was 2.60 me l^−1^. Na^+^ and K^+^ concentrations were determined using a Flame Photometer (Richards, 1954) and were 4.50 and 0.06 me l^−1^. Carbonate and bicarbonate concentrations were determined by titration with 0.01 N H_2_SO_4_ using phenolphthalein and methyl orange as indicators and chloride was determined through titration with 0.01 N AgNO_3_ solution using potassium chromate as indicator (Tandon, 1995, Richards, 1954). The respective concentrations were 1.0 me l^−1^, 4.0 me l^−1^ and 2.0 me l^−1^.

The climate of the experimental site is subtropical monsoon, with average annual rainfall of 817 mm. The highest rainfall during the cropping period (June–October) was received in August (331.8 mm), followed by July (215.5 mm), September (208.2 mm), and then June (167 mm). Total rainfall during 2011–2013 cropping seasons were 1034, 990 and 795 mm, respectively. The average annual evapotranspiration was 1580 mm, which varies with air temperature. The mean maximum temperature during the cropping period (July–October) was 33.5 °C in July and mean minimum temperature was 18.8 °C in October.

#### 2.2.2 Experimental details

Details of the two sets of experiments conducted during this study are presented below and summarized in Table 2. These experiments were conducted to develop matching management practices (Mmp) for CSR43 by optimizing existing management practices (Bmp) recommended for the Indo-Gangetic plains of Eastern Uttar Pradesh.

### 2.3 Optimizing number of seedlings hill^−1^ and spacing

To determine the optimum number of seedlings hill^−1^ and hill spacing for CSR43, a field experiment was conducted in 2011 with three replications. The main plots comprised two treatments, T_1_: 2 seedlings and T_2_: 4 seedlings per hill. Three spacing treatments were used as sub-plots; S_1_: 15 × 15 cm, S_2_: 15 × 20 cm, S_3_: 20 × 20 cm (Table 2). Thirty-day-old seedlings were transplanted in puddled and leveled field. Fertilizer rate at 120–60–40–25 kg N–P_2_O_5_–K_2_O–ZnSO_4_·7H_2_O ha^−1^ was applied uniformly. Half N and full P_2_O_5_, K_2_O and ZnSO_4_·7H_2_O were broadcasted as basal after puddling and the remaining N was applied in equal splits at active tillering (30 days after transplanting; DAT) and panicle initiation (60 DAT).

### 2.4 Optimizing nitrogen requirement in the field

To determine the optimum nitrogen application for transplanted CSR43 in these sodic soils, an experiment was conducted in 2013, with three replicates and six N treatments (N kg ha^−1^; N_1_: 0, N_2_: 100, N_3_: 125, N_4_: 150, N_5_: 175 and N_6_: 200) (Table 2). Thirty-day-old seedlings were transplanted at 4 seedlings per hill spaced at 15 × 20 cm. Full P_2_O_5_, K_2_O and Zn (60–40–25 kg N–P_2_O_5_–K_2_O–ZnSO_4_·7H_2_O ha^−1^) and half of N were applied as basal and the remaining N was applied in equal splits at active tillering (30 DAT) and panicle initiation (60 DAT).

In both experiments, seeds of CSR43 were treated with ceresin before sowing to control seed borne diseases. Nutrients at rates of 100–60–40 kg N–P_2_O_5_–K_2_O ha^−1^ along with 5 t FYM ha^−1^ were applied in the nursery, followed by seed sowing of 40 g m^−2^. Weeds were controlled using Butachlore (1.5 l ha^−1^) applied 2-3 DAT. No major disease or pest infestation was observed during 2011–2013.

### 2.5 Observations and data collection

After transplanting, 10 plants were tagged in each treatment and used for assessing growth and yield attributes at maturity. Plant height was measured from the base of the stem to the tip of the longest panicle at maturity. Days to maturity were determined when 80% of the spikelets within each treatment reached physiological maturity. Leaf area was measured using a leaf area meter (LAI-2200C, LI-COR) at the flag leaf stage and calculated using the formulae of Watson (1958). Panicles were hand threshed, filled and unfilled grains were separated manually and the number of spikelets per panicle was counted. After initial sun drying, grains and straw were dried in an oven (60–70 °C) to a constant weight and weighed. Floret fertility percentage, 1000-grain weight and harvest index (HI) were computed from collected data (Ying et al., 1998). To determine grain yield, grains were harvested from the net plot area of 20 m^2^, sun-dried and weighed. Grain moisture content was determined with a digital moisture meter (DMC-700, Seedburo, Chicago, IL, USA) and grain weight was adjusted to moisture content of 14%.

### 2.6 On-farm evaluation of the tolerant variety and best management practices

To validate the effect of management practices developed for CSR43 in on-station trials, on-farm evaluation was conducted at five farmers’ fields during the wet season of 2014 in Patwakhera village, Lucknow district, Eastern Uttar Pradesh, India (Table 2). Soil samples were collected and analyzed from 12 farmer’s fields, then five fields were selected with soil pH ranging from 9.0 to 9.5. Each farmer was considered one replicate with plot size of 300–400 m^2^. Four treatments were used; T_1_: Farmers’ varieties (Fv) with farmers’ management (Fm); T_2_: Improved variety (Iv, CSR43) with Fm, T_3_: Iv with existing best management practices (Bmp) recommended for sodic soils of Eastern Uttar Pradesh by ICAR-CSSRI; T_4_: Iv with matching management practices (Mmp) developed through on-station trials during 2011–2013. Grain yield was determined from an area of 20 m^2^.

### 2.7 Statistical and economic analyses

All data were subjected to analysis of variance (ANOVA) (Gomez and Gomez, 1984) using WINDOSTAT. The least significant difference (LSD) at 5% probability was used to compare treatment means (Steel and Torri, 1980). Data was presented as means across experiments for all parameters and main effects were reported as there are no significant interactions between plant spacing and number of seedlings hill^−1^. Economic analysis of different treatments was based on total production cost (fixed and variable), gross return, gross margin and benefit/cost ratio. Fixed costs included the cost of inputs (seed, fertilizers and FYM) and labor (field preparation, bunding, irrigation, fertilizers, harvesting and threshing), whereas, variable cost included labor charges for uprooting and transplanting of seedlings based on treatments in 2011 and varying rates of N and costs of application in 2013. Gross returns were calculated by multiplying the weight of grain by their corresponding minimum support price for respective year (US$ 190/t in 2011 and US$ 218/t in 2013) and market price of straw at harvest (16.66 US$/t during both years). The gross margin was calculated by subtracting total variable cost from gross returns, and the benefit/cost ratio was calculated by dividing gross return by total variable cost. The values were calculated using Indian rupees (INR), and then converted into US$ using conversion rate of 1US$ = 60 INR. Grain yield response to nitrogen was determined using curve expert model 32 and the economically optimum dose of N was calculated on the basis of maximum economic yield produced per unit of N (Fageria and Baligar, 2003).

## 3 Results

### 3.1 Evaluation and release of CSR43

After intensive evaluation and selection within the 126 genotypes from 2001 to 2004, only 6 breeding lines were selected (Table 1). Data on preference analysis and grain yield of these lines from a trial conducted in the 2005 wet season are presented in Table 3. Farmers ranked CSR-89IR-8, CSR36 and NDR359 as their first, second, and third preferred genotypes, respectively, with positive correlations between preference scores of male and female farmers (*r* = 0.91**) and between farmers and researchers (*r* = 0.74*). CSR-89IR-8 ranked first in all desired traits. CSR-2K-239 and CSR13 had the lowest scores for yield and other traits indicating they are not suitable for sodic soils. CSR36, and CSR-2K-262 showed high sodicity tolerance and grain yield but their duration is longer. Farmers selected CSR-89IR-8 as their preferred genotype because of several traits, including its good taste, aroma, color, non-cohesiveness when cooked and higher grain yield (Table 3). Moreover, the shorter duration of this variety saves 2–3 irrigations, allowed early establishment of rabi wheat; and its early maturity provided good market price (Singh et al., 2013, Singh et al., 2014).

### 3.2 Management options

#### 3.2.1 Optimizing number of seedlings hill^−1^ and spacing (Experiment I)

**Fig. 1:**
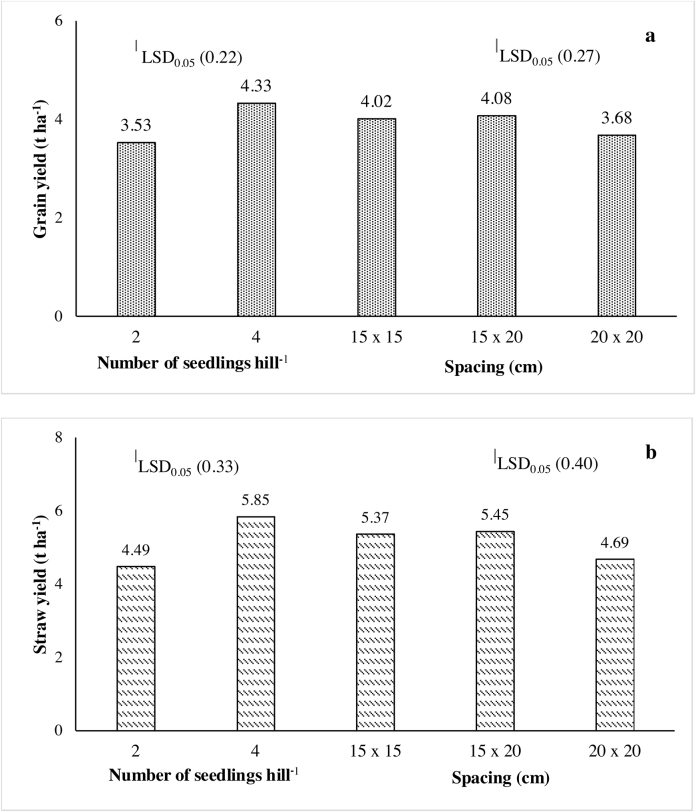
Grain (a) and straw (b) yields as influenced by number of seedlings hill^−1^ and spacing of transplanted CSR43 in on-station field trial conducted during 2011 wet season at ICAR-CSSRI research farm, Lucknow, India.

Growth, grain yield and yield attributes (except % fertility and 1000-grain weight) were significantly higher when 4 seedlings were transplanted per hill compared with 2 seedlings per hill (Table 4). Productive tillers hill^−1^, grain and straw yields with 4 seedlings hill^−1^ were, respectively, 15.9%, 22.7% and 30.3% higher than those with 2 seedlings hill^−1^ (Fig. 1a and b). Spacing had significant (*P* < 0.05) effect on plant height, fertility and grain yield. Using 15 × 20 cm spacing resulted in about 4–7% more spikelets panicle^−1^ over both 15 × 15 and 20 × 20 cm spacing though these differences were not significant. It also resulted in 11% higher grain yield and 16% higher straw yield over the values obtained with 20 × 20 cm spacing (Fig. 1a and b).

Planting of 4 seedlings hill^−1^ resulted in 23.4% and 42.6% higher gross and net returns, respectively, than planting of 2 seedlings hill^−1^ due to higher grain and straw yields. Transplanting of 4 seedlings hill^−1^ at a spacing of 15 × 20 cm significantly increased gross return, net return and BCR because of enhanced number of productive tillers and other yield attributes (Table 5). Fixed cost (329.5 US$ ha^−1^) incurred in this experiment is presented as Supplemental Table 1.

#### 3.2.2 Optimizing N requirements for sodic soils (Experiment II)

Plant height and productive tillers hill^−1^ increased with increasing N application (Table 6). Application of 200 kg N ha^−1^ produced 70%, 53.8%, 21.5%, 9.7% and 3.5% more productive tillers hill^−1^ than the application of 0, 100, 125, 150 and 175 kg N ha^−1^, respectively. Maximum leaf area index (LAI) was attained with 200 and 175 kg N ha^−1^ and was lowest in the control treatment. Increasing nitrogen extended maturity duration because of prolonged vegetative growth (Table 6).

**Fig. 2:**
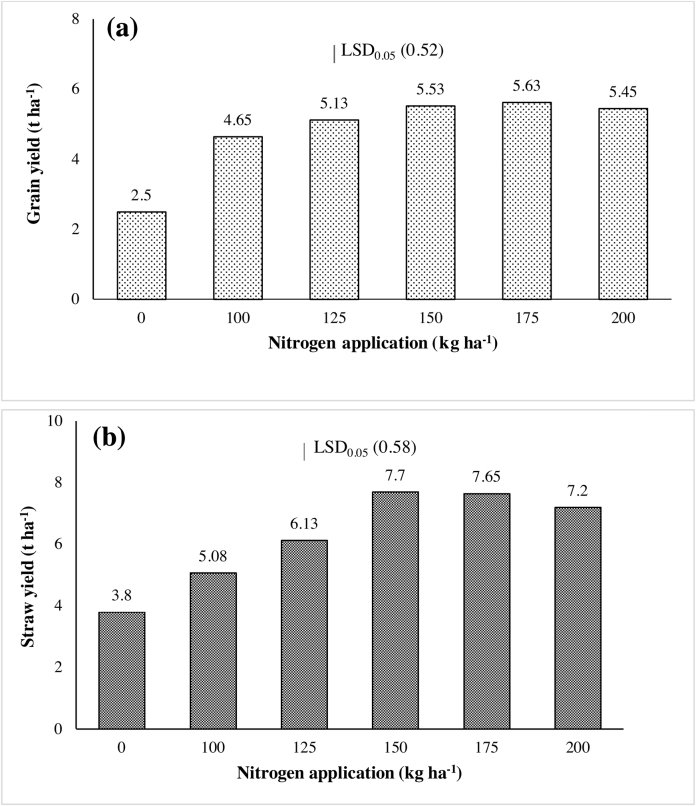
Grain (a) and straw (b) yields of CSR43 as influenced by N treatments in an on-station trial conducted during 2013 wet season at ICAR-CSSRI research farm, Lucknow, UP, India.

**Fig. 3:**
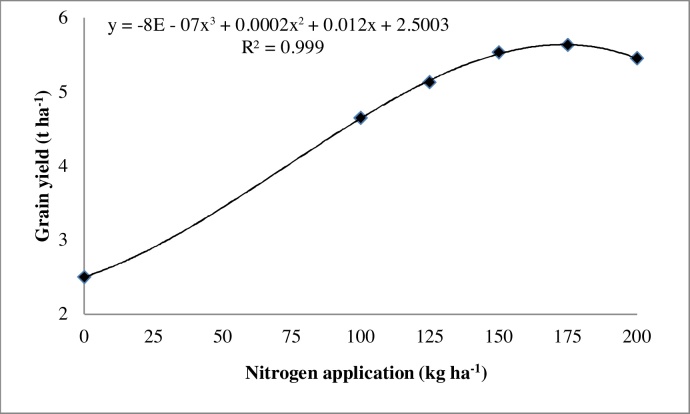
Trend analysis of grain yield of the rice variety CSR43 as affected by different N treatments in an on-station field trial conducted during the 2013 wet season at ICAR-CSSRI research farm, Lucknow, UP, India.

Number of spikelets panicle^−1^ and 1000-grain weight increased significantly with increasing N to 200 and 175 kg ha^−1^ (Table 6). Applying 100 kg N ha^−1^ resulted in 125% increase in grain yield over the control (Fig. 2a). Grain yield was statistically similar when 175, 200 and 150 kg N ha^−1^ were used, but these yields were significantly higher than when 0, 100 and 125 kg of N were applied. Similar pattern was also observed for straw yield (Fig. 2a and b). Trend analysis using the curve model “Expert 32” (Danial, 1995) indicated that grain yield progressively increased with increasing N application, peaking at 169 kg ha^−1^. Afterwards, grain yield declined with further increase in N application (Fig. 3).

**Fig. 4:**
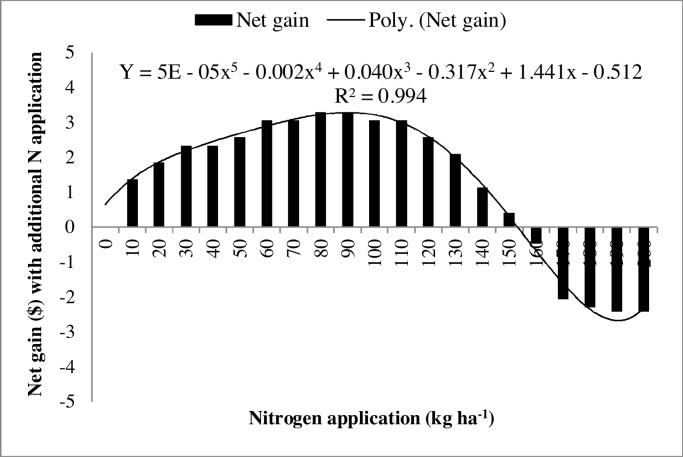
Analyses of the economic optimum amount of N for rice variety CSR43 under sodic field conditions.

Production cost increased with increasing N application due to higher variable costs (0.25 US$/kg) and higher labor charges (2.5 US$/8 h) for its application (Table 7). Application of 175 kg N ha^−1^ resulted in the highest gross and net returns, while 150 kg N ha^−1^ resulted in the highest BCR, however, differences between 150 and 175 kg N treatments were not significant. Further increase in N application to 200 kg ha^−1^ resulted in significant reduction in gross return, net return, and BCR. The BCR recorded with 150 kg N ha^−1^ was 78% and 15% higher than zero N (control) and 200 kg N ha^−1^ (Table 7). Returns from incremental increase in N application were analyzed to determine the economically optimum amount. This was estimated by analyzing the additional net gain from the application of additional N over the range of nitrogen treatments. The additional net gain of each incremental N dose was calculated by subtracting the net gain of the preceding incremental N dose from the net gain of the succeeding dose. The highest net additional gain was US$ 3.3 at 90 kg ha^−1^ N, and the lowest net additional gain was US$ 0.4 at 150 kg ha^−1^ N (Fig. 4). Above 150 kg ha^−1^, the net additional gain became negative, indicating decreasing returns from additional N application. Hence, 150 kg N ha^−1^ was considered the economical optimum for CSR43 in these sodic soils. The polynomial 5^th^ order equation effectively explained the net gain against corresponding nitrogen treatments (*R*^2^ = 0.994; Fig. 4).

### 3.3 Grain yield of the salt tolerant variety CSR43 as affected by different management packages in on-farm trials

**Fig. 5:**
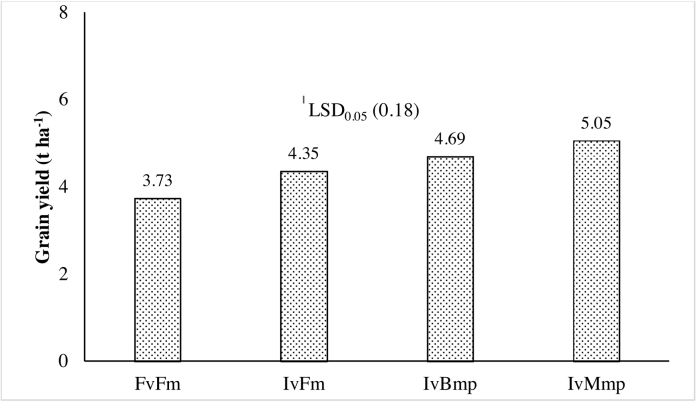
Grain yield as influenced by varieties and management practices in on-farm trials in Eastern Uttar Pradesh during 2014 wet season. FvFm, Farmer’s variety with farmer’s management; IvFm, improved variety with farmer’s management; IvBmp, improved variety with best management practices (existing); IvMmp, improved variety with matching management practices developed in this study for CSR43. Farmers’ varieties were Ganga Kaveri, Moti and Narendra359.

The set of best management practices established for CSR43 in the above trials was then validated in 5 farmers’ fields during the wet season of 2014 (Table 2). The selected matching management practices (Mmp) included using 4 seedlings per hill at 15 × 20 cm spacing and application of fertilizers in the field at 150–60–40–25 kg N–P_2_O_5_–K_2_O–ZnSO_4_.7H_2_O ha^−1^. Grain yield varied significantly between the four treatments (Fig. 5). The highest grain yield of CSR43 (Iv) was when using the new package of Mmp, which resulted in 8% higher yield than the treatment using existing best management practices (Bmp) recommended for the region (Table 2). Moreover, the combination of IvMmp treatment resulted in 35% and 16% higher grain yield over FvFm and IvFm, respectively. Ganga Kaveri, Moti and Narendra359 rice varieties were used to represent farmers’ varieties (Fv), and neither produced higher yields mainly because of their relatively lower tolerance of salt stress and poor management practices followed by farmers when using FvFm (Table 2). However, under the same farmers’ management (Fm), yield of CSR43 was 17% higher than the other varieties (IvFm vs FvFm) (Fig. 5).

## 4 Discussion

### 4.1 Selection and release of CSR43 as salt tolerant variety through FPVS

The FPVS program conducted in sodic soils (Paris et al., 2011) has confirmed the relevance and utility of participatory approaches in these less favorable environments. On-station screening of a large number of genotypes helped to narrow the number of breeding lines suitable for sodic soils. Studies on farmers’ preferences (Table 3) revealed that, farmers generally preferred salt tolerant, short duration varieties that are suited for the rice: wheat or rice: legume: wheat rotations. Moreover, the shorter stature of CSR-89IR-8 made it relatively tolerant to lodging at maturity, causing less yield losses. Based on farmers’ ranking, CSR-89IR-8 was most preferred. These attributes together culminated in the release of this breeding line as CSR43 in 2011 (Singh et al., 2013). The early maturity of CSR43 further saves two irrigations compared with other medium to longer duration varieties (Singh et al., 2014); this saving of irrigation water increased BCR and early harvest can benefit subsequent rabi season crops. Singh et al. (2014) further observed that in the selected villages where FPVS was conducted, the adoption of CSR43 among farmers increased remarkably year after year. In contrast, the adoption rate of a popular variety ‘Narendra359’, which has been recommended for normal high yielding environments in the region, declined by one fourth over the previous year in sodic areas (Singh et al., 2014).

### 4.2 Developing matching management options

#### 4.2.1 Effect of seedlings hill^−1^ and spacing during transplanting on crop growth and yield

Grain yield was significantly enhanced with transplanting of four seedlings hill^−1^ under medium spacing (15 × 20 cm). Higher number of seedlings hill^−1^ resulted in significantly higher dry matter production and more productive tillers hill^−1^, whereas medium spacing enhanced floret fertility resulting in higher grain yield over other treatments. The production of more tillers in widely spaced plants is usually explained by the availability of more nutrients, moisture and sunlight as compared with dense planting. This was also reported by Shieh (1979); Ayub et al. (1987); Miah et al. (1990) and Prasad et al. (1992). Karmakar et al. (2002) observed that panicles became gradually shorter with increasing number of seedlings hill^−1^. These results indicate the need for optimizing crop management practices such as number of seedlings hill^−1^ and spacing between hills to attain optimum plant growth, canopy development and yield formation for specific soil conditions and variety.

#### 4.2.2 Effect of nitrogen applied in the field on crop growth and yield of transplanted CSR43

N application is necessary to maintain plant growth and enhance grain yield. The highest dose of 200 kg N ha^−1^ produced the tallest plants and the highest number of productive tillers. Behra (1998) and Dwivedi et al. (2000) also reported significant increase in plant height and number of tillers with increasing N application. Similar beneficial effects of N application in similar environments were reported before (Mandal et al., 1994, Singh et al., 1990, Assy et al., 1992, Singh and Sharma, 1993, Chaturvedi, 2005, Manzoor et al., 2006, Nawaz, 2002). Grain yield of CSR43 responded positively to N application up to 169 kg ha^−1^ (Fig. 3), however, gross margin and BCR were highest with 150 kg N ha^−1^ (Fig. 4; Table 7). This N rate can therefore, be recommended for CSR43 in these sodic soils. This is 25% higher than the recommended dose of N (120 kg N ha^−1^) for farmers in this region. Responses of rice to higher N in sodic soils was also reported before (Singh and Sharma, 1984, Singh, 1987, Dixit and Patro, 1994, Meena et al., 2003).

### 4.3 On-farm evaluation of CSR43 using matching management options

CSR43 had on average, a yield advantage of 0.62 t ha^−1^ over farmers’ varieties across locations (range of 0.39–0.82 t ha^−1^). However, combined effect of improved variety with matching management practices resulted in a 35% increase in grain yield over farmers’ current practices. The average yield enhancement due to these best management practices across locations was 16%. Studies by Ismail et al. (2012) highlighted the benefits of combining good management practices with genetic tolerance for better crop establishment in less favorable areas, as tolerant genotypes respond better to these agronomic manipulations than sensitive ones. Purushottam et al. (2012) reported that grain yield of a rice–wheat system in on-farm trials did not increase significantly with the use of improved varieties alone, but a significant increase over farmers’ practices was observed when improved varieties were combined with line sowing/transplanting. Sarangi et al. (2015) also reported higher yields when farmers use improved varieties with improved nursery management practices. Our study highlighted the importance of developing and validating management options for new salt tolerant varieties to maximize their attainable yields in farmers’ fields.

## 5 Conclusions

CSR43 produced about 0.5 t ha^−1^ additional grain yields over farmers’ current varieties across locations. This yield advantage, together with tolerance of sodicity, short duration, good grain quality and market value contributed to the selection and commercialization of this variety and to its faster adoption in the sodic soils of the Indo-Gangetic plains (Singh et al., 2014). Matching crop establishment and nitrogen rates were developed for this variety, and when combined, resulted in significantly higher yields and cost/benefit ratios compared with current varieties and agronomic practices used by local farmers. Suggested recommendations include transplanting of 4 seedlings hill^−1^ at a spacing of 15 × 20 cm and use of 150–60–40–25 kg N–P_2_O_5_–K_2_O–ZnSO_4_·7H_2_O ha^−1^ in the field. On-farm validation trials of these practices using CSR43 showed that the matching management practices alone increased grain yield by 8% over existing recommendations and by 16% over management strategies being practiced by local farmers. This supports our hypothesis that matching crop and nutrient management practices are essential for harnessing the full potential of a new stress-tolerant variety in targeted conditions. These cost effective management options for sodic soils can further be refined to match other new tolerant varieties as they become available. The benefits can then be extended to larger areas including the salt-affected areas of the Ganges mega deltas in India and Bangladesh, in addition to the Indo-Gangetic plains of Eastern Uttar Pradesh and Bihar in India.

## Figures and Tables

**Table 1:** Processes followed in the selection of CSR43 through farmers’ participatory varietal selection.

Year	Process	Number of genotypes planted in sodic soil	Number of genotypes selected	Number of trial sites in sodic soils per year
2001	Screening of genotypes (by breeders)	126	18	2
2002	FPVS	18	12	5
2003	FPVS	12	9	6
2004	FPVS	9	6	6
2005	Final selection through FPVS	6	2	6
2006–2008	Evaluation through farmers managed trials	2 new + 1 farmers variety	1 selected; released as “CSR43” in 2011	32

**Table 2:** Details of field experiments conducted at ICAR-CSSRI, RRS, Lucknow (on-station) to develop management practices for CSR43 during the wet seasons of 2011 and 2013 and validation in farmers’ fields (on-farm) during 2014 wet season.

Experiment and design	Treatments	Treatment details
On-station trials (wet seasons 2011–2013)		
Experiment I		
Optimizing number of seedlings hill^−1^ and spacing	Main plot: seedlings hill^-1^ (T)	T_1_: 2, T_2_: 4
(Split-plot)	Sub-plot: spacing (S)	S_1_: 15 × 15 cm, S_2_: 15 × 20 cm, S_3_: 20 × 20 cm
Experiment II		
Nutrient management (RBD)	Nitrogen level (N)	N_1_: 0, N_2_: 100, N_3_: 125, N_4_: 150, N_5_: 175, N_6_: 200 kg ha^−1^; 60–40–25 kg P_2_O_5_–K_2_O–ZnSO_4_.7H_2_O ha^−1^ applied to all plots

On-farm experiments (wet season, 2014)		
Validation of management practices for CSR43 (RBD)	FvFm	1–2 seedlings hill^−1^, random planting, and 100–0–0 kg N–P_2_O_5_–K_2_O ha^−1^; varieties: Ganga Kaveri, Moti and Narendra359
IvFm	Management practices same as above except using variety CSR43
IvBmp	3 seedlings hill^−1^ at 15 × 15 cm, and recommended fertilizer application of 120–60–40–25 kg N–P_2_O_5_–K_2_O–ZnSO_4_·7H_2_O ha^−1^ in main field; variety: CSR43
IvMmp	4 seedlings hill^−1^ at 15 × 20 cm, with fertilizer application of 150–60–40–25 N–P_2_O_5_–K_2_O–ZnSO_4_·7H_2_O ha^−1^ in main field; variety: CSR43

FvFm: farmers’ variety and farmer management; IvFm: improved variety and farmers’ management; IvBmp: improved variety and existing (best) management practices; IvMmp: improved variety and improved (matching) management practices established in on-station experiments.

**Table 3:** Preference scores^a^ (mean ± SE) of desirable traits and on-station and on-farm grain yield of 6 rice genotypes evaluated during the 2005 wet season at Lucknow, UP, India.

Traits	CSR-2K-239	NDR359	CSR13	CSR-89IR-8	CSR36	CSR-2K-262
	(*n* = 42)	(*n* = 42)	(*n* = 42)	(*n* = 42)	(*n* = 42)	(*n* = 42)
Plant height	6.0 ± 0.5	6.0 ± 0.4	6.0 ± 0.4	8.0 ± 0.5	6.0 ± 0.5	7.0 ± 0.4
Productive tillers hill^−1^	6.0 ± 0.5	7.0 ± 0.4	6.0 ± 0.6	8.0 ± 0.5	8.0 ± 0.8	7.0 ± 0.8
Days to maturity	6.0 ± 0.4	6.0 ± 0.6	7.0 ± 0.5	9.0 ± 0.4	7.0 ± 0.6	7.0 ± 0.7
Lodging tolerance	6.0 ± 0.7	6.0 ± 0.5	6.0 ± 0.6	8.0 ± 0.6	7.0 ± 0.7	7.0 ± 0.5
Sodicity tolerance	6.0 ± 0.7	7.0 ± 0.8	7.0 ± 0.7	8.0 ± 0.5	8.0 ± 0.6	8.0 ±n0.7
Grain quality	6.0 ± 0.7	7.0 ± 0.7	7.0 ± 0.7	8.0 ± 0.7	7.0 ± 0.7	8.0 ± 0.8
Cooking quality	6.0 ± 0.7	6.0 ± 0.7	6.0 ± 0.8	7.0 ± 0.8	7.0 ± 0.8	7.0 ± 0.8

Grain yield (t ha^−1^)						
On-station	4.9 ± 0.2	5.0 ± 0.4	5.0 ± 3.0	5.2 ± .04	5.1 ± 0.4	5.1 ± 0.3
On-farm	4.8 ± 0.3	4.9 ± 0.2	4.9 ± 0.3	5.1 ± 0.4	5.1 ± 0.3	5.0 ± 0.3
Preference score	−0.05	0.12	0.09	0.20	0.18	0.04

a Preference scoring scale (1–10), 1 = least preferred; 10 = most preferred.

**Table 4:** Effect of number of seedlings hill^−1^ and spacing on growth of transplanted CSR43 in an on-station field trial in 2011 wet season at ICAR-CSSRI research farm, Lucknow, UP, India.

Treatments	Plant height (cm)	Productive tillers hill^−1^	Dry matter (g hill^−1^)	No. of spikelets panicle^−1^	Fertility (%)	1000-grain weight (g)
Number of seedlings hill^−1^						
2	98.3	9.4	52.0	125	85.7	25.9
4	96.7	10.9	63.7	143	84.2	25.0
LSD_0.05_	ns^a^	1.2	2.6	7	ns	ns

Spacing (cm)						
15 × 15	97.8	9.3	56.8	129	84.9	24.8
15 × 20	98.9	11.1	58.6	138	87.8	25.3
20 × 20	95.7	10.1	58.2	133	82.3	26.3
LSD_0.05_	1.9	ns	ns	ns	3.36	ns

a ns, not significant.

**Table 5:** Economic indicators of different crop establishment options in on-station field trial conducted during 2011 wet season at ICAR-CSSRI research farm, Lucknow, UP, India.

Treatments	Variable cost(US$ ha^−1^)		Production cost (fixed^a^ + variable) (US$ ha^−1^)	Gross return (US$ ha^−1^)	Net return (US$ ha^−1^)	BCR^b^
	Uprooting	Transplanting				
Number of seedlings hill^−1^						
2	12.3	24.9	367	745	378	2.03
4	16.7	33.7	380	919	539	2.41
LSD_0.05_	–	–	–	47	47	0.12

Spacing (cm)						
15 x 15	16.7	33.7	380	853	473	2.24
15 x 20	14.5	29.3	373	867	494	2.32
20 x 20	12.3	24.8	367	776	409	2.11
LSD_0.05_	–	–	–	58	58	0.15

a Fixed cost (329.5 US$ ha^−1^).

b BCR, benefit/cost ratio.

**Table 6:** Effect of N treatments on growth and yield attributes of CSR43 in an on-station field trial conducted during 2013 wet season at ICAR-CSSRI research farm, Lucknow, UP, India.

N level (kg ha^−1^)	Plant height (cm)	Productive tiller hill^−1^	LAI	Days to maturity (d)	Number of spikelets panicle^−1^	Fertility (%)	1000-grain weight (g)
0	96	8.6	4.6	105	83	89.6	24.6
100	100	9.1	5.2	108	111	85.3	27.6
125	103	12.1	5.4	110	121	88.6	26.7
150	106	13.4	5.6	112	134	92.0	28.1
175	106	14.2	6.0	115	142	90.0	28.1
200	108	14.7	6.1	120	145	91.0	28.4
LSD_0.05_	ns^a^	1.2	0.1	3.2	8.7	ns	1.05

a ns, not significant.

**Table 7:** Economic indicators of different N treatments in an on-station field trial conducted during the wet season of 2013 at ICAR-CSSRI research farm, Lucknow, UP, India.

N levels (kg ha^−1^)	Production cost (US$ ha^−1^)	Gross return (US$ ha^−1^)	Net return (US$ ha^−1^)	BCR^a^
0	313	600	289	1.93
100	359	1101	742	3.07
125	371	1221	850	3.29
150	383	1318	935	3.44
175	395	1339	944	3.39
200	408	1211	803	2.97
LSD_0.05_	–	115	115	0.16

a BCR benefit/cost ratio; selling price of grain was 218 US$/t, and straw was 16.7 US$/t.
