# Supplementary material for: Productivity of sodic soils can be enhanced through the use of salt tolerant rice varieties and proper agronomic practices
Source: Field Crops Res. 2016 Apr;190:82–90. doi: 10.1016/j.fcr.2016.02.007 (PMC4862441; doi:10.1016/j.fcr.2016.02.007)
Supplement: Supplementary file 1 [file mmc1.docx]

**Supplemental Table 1.**

Fixed cost (US$) incurred in experiment 1 on inputs and operations during the 2011 wet season at ICAR-CSSRI research farm, Lucknow, UP, India.

| Particular | | | | | | Time/quantity | Rate (US$) | Cost (US$) |
| --- | --- | --- | --- | --- | --- | --- | --- | --- |
| **Nursery** | | | | | | | | |
| Field preparation (1000 m^2^) | 2 harrowing + 1 cultivator and planking (mechanical) | | | | | 0.4 h | 4.16 hr^-1^ | 2.77 |
| Seed | Purchase | | | | | 40 kg | 0.50 kg^-1^ | 20 |
|  | Treatment (Ceresin) | | | | | 100 g | 16 kg^-1^ | 1.6 |
|  | Sowing | | | | | 4 h | 0.25 hr^-1^ | 1.0 |
| Fertilizer (100-60-40-25 kg N-P_2_O_5_-K_2_O-ZnSO_4_.7H_2_O ha^-1^) | Cost | | N | | | 10 kg | 0.225 kg^-1^ | 9.22 |
|  |  |  | P_2_O_5_ | | | 6 kg | 0.58 kg^-1^ |  |
|  |  |  | K_2_O | | | 4 kg | 0.46 kg^-1^ |  |
|  |  |  | ZnSO_4_.7H_2_O | | | 2.5 kg | 0.66 kg^-1^ |  |
|  | Application | | | | | 2 h | 0.25 hr^-1^ | 0.50 |
| [FYM 5 (t ha](mailto:FYM@5%20t/ha)^-1^) |  | | | | | 0.5 t | 8.33 t^-1^ | 4.16 |
| Irrigation (6) | Labour | | | | | 12 h | 0.25 hr^-1^ | 3.0 |
|  | Machine | | | | | 6 h | 0.83 hr^-1^ | 5.0 |
| **Main field** | | | | | | | | |
| Field preparation | 1 harrow +1 cultivator + 1 puddling and planking (mechanical) | | | | | 8 h | 4.16 hr^-1^ | 33.33 |
| Irrigation (5) | Labor | | | | | 80 h | 0.25 hr^-1^ | 20.0 |
|  | Machine | | | | | 45 h | 0.83 hr^-1^ | 37.5 |
| Fertilizer (120-60-40-25 kg N-P_2_O_5_-K_2_O-ZnSO_4_.7H_2_O ha^-1^) | Cost | | | N | | 120 kg | 0.225 kg^-1^ | 96.7 |
|  |  |  |  | P_2_O_5_ | | 60 kg | 0.58 kg^-1^ |  |
|  |  |  |  | K_2_O | | 40 kg | 0.46 kg^-1^ |  |
|  |  |  |  | ZnSO_4_.7H_2_O | | 25 kg | 0.66 kg^-1^ |  |
|  | Application | | | | | 4 h | 0.25 hr^-1^ | 1.0 |
| Weeding | Hand | | | | | 40 h | 0.25 hr^-1^ | 10.0 |
|  | Chemical (Butachlore) | | | | | 1.5 liter | 2.5 L^-1^ | 3.75 |
| Plant protection | No insecticide and pesticide used | | | | | | | |
| Harvesting |  | | | | | 160 h | 0.25 hr^-1^ | 40.0 |
| Threshing |  | | | | | 160 h | 0.25 hr^-1^ | 40.0 |
| Transportation | Auctioned at site | | | | | | | |
| Total |  |  | | |  |  |  | **329.5** |

Labour: 2 US$/8hr, Tractor: 4.16 US$/hr, Seed: 0.50 US$/kg, Tube well: 0.83 US$/h, N: 0.225 US$/kg, P_2_O_5_: 0.58 US$/kg, K_2_O: 0.46 US$/kg, ZnSO_4_.7H_2_O: 0.66 US$/kg, Herbicide: 2.5 US$/L, FYM: 8.33 US$/t, price of grain: 190 US$/t; price of straw: 16.66 US$/t.
